# 基于代谢组学和质谱成像法考察烟碱暴露对小鼠脑内内源性代谢物的影响

**DOI:** 10.3724/SP.J.1123.2024.10005

**Published:** 2025-04-08

**Authors:** Lulu GUO, Chen ZHANG, Yanjun HUANG, Xingyu LIU, Deshui LIU, Teng LONG, Jinhao SUN, Shaofeng LIU, Zhonghao LI, Jiazhong WANG, Jian MAO

**Affiliations:** 1.北京生命科技研究院有限公司,北京 102209; 1. Beijing Life Science Academy Co., Ltd., Beijing 102209, China; 2.中国农业大学营养与健康系, 北京 100193; 2. Department of Nutrition and Health, China Agricultural University, Beijing 100193, China; 3.贵州中烟工业有限责任公司技术中心,贵州 贵阳 550009; 3. Technology Center, China Tobacco Guizhou Industrial Co., Ltd., Guiyang 550009, China

**Keywords:** 超高效液相色谱-串联质谱, 代谢组学, 质谱成像, 烟碱, 鼠脑, ultra-high performance liquid chromatography-tandem mass spectrometry (UHPLC-MS/MS), metabolomics, mass spectrometry imaging, nicotine, mouse brain

## Abstract

烟碱具有明显的神经药理学活性,对多种中枢神经系统疾病具有干预作用,然而烟碱入脑后对脑区内源性代谢物的影响尚不清楚。研究烟碱暴露后对小鼠脑内内源性代谢物的影响,可为阐明烟碱的多重生理学效应提供更加全面的物质基础。本文基于超高效液相色谱-串联质谱(UHPLC-MS/MS)及代谢组学分析方法,系统考察了烟碱多次暴露后对小鼠脑内内源性代谢产物的影响,并进一步采用质谱成像法,直观获取烟碱引发的差异性代谢物在不同脑区的分布及变化差异。实验结果如下:本研究共鉴定到759种代谢物,烟碱暴露后共筛选到575个显著差异性代谢物,其中434个代谢物下调,141个代谢物上调;通过京都基因和基因组数据库(Kyoto Encyclopedia of Genes and Genomes, KEGG)通路富集分析表明烟碱暴露后主要影响到脑内必需氨基酸代谢、脂质代谢、核苷酸代谢、碳水化合物代谢、辅因子和维生素代谢以及其他氨基酸代谢通路;进一步通过质谱成像法分析差异性代谢物在脑内的分布特征,结果表明烟碱暴露后全脑范围内胆碱、丝氨酸、天冬氨酸及苹果酸含量都被显著下调,皮层区域牛磺酸、海马区域乙酰基肉碱以及纹状体区域的腺苷类物质均被显著影响。这些结果为进一步揭示烟碱暴露对中枢神经系统的影响提供了新的实验依据。

## Abstract (en)

Nicotine, the principal alkaloid in tobacco, exhibits significant central nervous system activity and induces a wide array of physiological effects. In addition to its well-documented role in tobacco dependence, previous studies have suggested that nicotine also has diverse pharmacological properties. These include alleviating symptoms associated with Parkinson’s disease, potentially reducing the risk of Alzheimer’s disease, mitigating oxidative stress, as well as anti-inflammatory and anxiolytic effects. Neuroscientists frequently use an array of molecular biology techniques to elucidate the mechanisms responsible for the effects of nicotine on the central nervous system. However, disease onset is invariably accompanied by metabolic dysfunction, and organisms often exhibit complex and unpredictable responses to pharmacological stimuli. As a bioactive alkaloid with potent pharmacological properties, nicotine is able to cross the blood-brain barrier and induce brain-compound changes, which serves as the basis for its effects on the central nervous system. Consequently, examining the extensive impact of nicotine exposure on endogenous metabolites and metabolic pathways in the brain is an indispensable step toward providing a more robust foundation for understanding the complex physiological effects of nicotine.

In this study, an ultra-high performance liquid chromatography-tandem mass spectrometry (UHPLC-MS/MS) metabolomic-analysis method was established to systematically examine the effects of repeated nicotine exposure on endogenous metabolites in mouse brains. Two chromatographic systems fitted with Acquity UPLC BEH HILIC (150 mm×2.1 mm, 1.7 μm) and BEH C18 (150 mm×2.1 mm, 1.7 μm) columns were used to determine the nicotine present in samples. As a result, the established UHPLC-MS/MS method identified a total of 759 endogenous metabolites. Compared with the saline group, nicotine exposure resulted in 575 significantly different metabolites, with 434 metabolites down-regulated and 141 up-regulated. Further pathway-enrichment analysis using the Kyoto Encyclopedia of Genes and Genomes (KEGG) revealed that nicotine exposure primarily affects essential-amino-acid, lipid, nucleotide, carbohydrate, cofactor, and vitamin metabolism, as well as other amino-acid metabolic pathways in the brain. Although non-targeted metabolomics can simultaneously detect and analyze all small-molecule metabolites in an unbiased manner, accurately capturing metabolite changes in specific brain regions is challenging when dealing with complex brain-tissue systems. Targeting the aggregation of material bases and the delivery of precision treatment to certain brain regions is expected to be significant for the targeted therapy of central nervous system diseases. Airflow-assisted desorption electrospray ionization mass spectrometry imaging (AFADESI-MSI) was further used to directly visualize the nicotine-induced distributions and variations of differentially expressed metabolites in various brain regions, which revealed that nicotine exposure leads to the significant downregulation of choline, serine, aspartate, and malate levels throughout the brain. Specifically, taurine, acetylcholine, and adenosine levels were notably affected in the cortical, hippocampal, and striatal regions, respectively. Essential-amino-acid metabolism was most affected by nicotine, with lipid metabolism found to be the next-most affected pathway. These metabolic pathways predominantly affected the cortical region, whereas the striatum, hippocampus, thalamus, and cerebellum were affected to varying degrees. These findings provide novel experimental evidence that enhances our understanding of metabolic biomarkers associated with nicotine exposure.

烟碱(nicotine)是烟草中的主要生物碱,具有明显的中枢神经活性,可引发多重生理学效应。烟碱不仅与烟草依赖的发生有关,既往研究发现其还具有缓解帕金森病症状^[1⇓⇓-4]^、降低阿尔兹海默发病风险^[5⇓-7]^、减轻氧化应激^[8]^及抗炎抗焦虑^[9,10]^等多种神经药理学作用。在神经科学领域,研究者通常采用多种分子生物学手段,从烟碱对脑内烟碱型乙酰胆碱受体调节、烟碱对不同脑区间神经元连接及调控、烟碱对脑内胶质细胞活性调节以及烟碱对炎症相关因子的释放影响等角度阐释其对中枢神经系统的干预机制^[11]^。然而,疾病的发生必然伴随着机体代谢功能紊乱,生物体通常以复杂且不可预测的方式对药物刺激做出反应,烟碱作为一种具有强药理活性的生物碱,透过血脑屏障引发脑内化合物发生改变是其在中枢神经系统发挥作用的物质基础。因此研究烟碱暴露后对脑内内源性代谢物及代谢通路的整体影响,将为揭示烟碱干预神经系统的作用机制提供新的依据。

代谢组学通过对生物样本中内源性小分子代谢物进行定性和半定量分析,研究代谢物和代谢通路的改变,为寻找疾病有关生物标志物和预测药物对疾病的干预机制提供关键信息和科学研究基础^[12⇓-14]^。非靶向代谢组学能无偏向地对所有小分子代谢物进行同时检测分析,但在面对复杂的脑组织体系时,难以准确获取特定脑区代谢物的改变。如果将物质基础的聚集与治疗精准定位到某些脑分区,将对中枢神经系统疾病的靶向治疗具有重要的意义。

质谱成像(mass spectrometry imaging, MSI)技术作为近年来的质谱领域的研究前沿和热点方法,是通过对样品中的数百种化学分子按照空间位置逐点扫描采集,同时获取化合物的离子强度与空间位置信息,然后通过数据处理软件对*m/z*、响应强度及空间位置信息进行三维重构,直观获取靶标物质的空间分布及含量信息,为理解生命活动和药物作用的分子机制提供了直观准确的分析手段^[15,16]^。近年来,空气动力辅助解吸电喷雾离子化质谱成像(AFADESI-MSI)技术在体内药物原位表征与作用机制、肿瘤生物标志物的原位筛选以及免标记分子病理诊断研究中被广泛应用^[17⇓-19]^。

本研究建立了超高效液相色谱-串联质谱(UHPLC-MS/MS)方法,采用亲水相互作用色谱(HILIC)柱和反相(C18)柱对非极性和极性化合物进行全面分析,系统考察了烟碱多次暴露对小鼠脑内内源性代谢物的影响。进一步通过质谱成像技术,对代谢组获取的差异性代谢物进行了小鼠脑内的空间分布研究,为阐释烟碱的多重神经药理学效应机理提供了新的实验依据。

## 1 实验部分

### 1.1 仪器和试剂

代谢组学分析使用Thermo Fisher DIONEX Ultimate 3000超高效液相色谱仪和Thermo Fisher Q-Exactive HF-X质谱仪;质谱成像分析使用空气动力辅助解吸电喷雾离子化质谱成像系统(AFADESI-MSI,北京维科托科技有限公司)和Thermo Fisher Orbitrap Exploris 240质谱仪。Compound Discoverer 3.3.1软件(Thermo, USA); Leica CM3050 S冰冻切片机(Leica Biosystems, Germany);ST2R PLUS台式高速冷冻离心机(Thermo Fisher, USA);组织匀浆机(JX-FSTPRP-48,上海净信实业发展有限公司);IKA VIBRAX VXR BS25涡旋混匀仪(IKA, Germany)。

烟碱购自多伦多研究化学(TRC, Canada,纯度>99%),质谱级甲醇和乙腈均购自Fisher Scientific (USA);色谱级甲酸购自Sigma-Aldrich (USA),试验用水为屈臣氏水。

### 1.2 样品制备

#### 1.2.1 实验动物

选用C57BL/6成年雄性小鼠(8~12周龄),体重18~22 g(北京维通利华生物技术有限公司),小鼠至少提前一周饲养在12 h光照和12 h黑暗交替循环的动物房中(早晨8时开灯,晚上20时关灯),温度22~26 ℃,湿度40%~60%,所有小鼠均可以自由获取食物和水。所有实验程序均经中国农业大学实验动物福利与实验动物伦理审查委员会批准并按照标准操作进行,批准文号为AW31012202-4-2。依据实验组别,将小鼠随机分笼并标记为盐水组和烟碱组。

#### 1.2.2 脑匀浆样品制备

实验用小鼠14只,随机分为2组,分别为盐水组和烟碱组。烟碱组每天腹腔注射1 mg/kg烟碱(0.1 mg/mL, 200~220 μL/只),对照组每天注射200 μL生理盐水,连续注射2周,在最后一次注射完成24 h后取脑,称重。按照20 mg组织加入800 μL 80%甲醇水溶液,1800 r/min下匀浆3 min,每匀浆20 s间隔10 s,制备脑匀浆液,于12000 r/min, 4 ℃下离心10 min,收集上清液,室温下进行N_2_吹干后采用100 μL乙腈复溶,再次离心,取上清液于色谱瓶中备用。质量控制(QC)样品为所有脑匀浆的混合样品。

#### 1.2.3 质谱成像样品制备

动物处理方法同上。在最后一次注射完烟碱24 h后取出脑组织,液氮速冻后,使用冷冻切片机进行脑组织切片,切片厚度10 μm,转移至载玻片上,-80 ℃保存用于成像分析。

### 1.3 仪器分析条件

#### 1.3.1 代谢组学-UHPLC-MS/MS方法

HILIC条件 样品分离使用Acquity UPLC BEH HILIC柱(150 mm×2.1 mm, 1.7 μm, Waters, USA);柱温为40 ℃,流速为0.3 mL/min, A相为0.1%的甲酸水溶液,B相为0.1%的甲酸乙腈溶液;流动相梯度洗脱条件:0~0.5 min, 98%B; 0.5~10 min, 98%B~60%B; 10~16 min, 60%B; 16~18 min, 60%B~98%B; 18~20 min, 98%B。进样量为1 μL。

反相色谱条件 Acquity UPLC BEH C18柱(150 mm×2.1 mm, 1.7 μm, Waters, USA);柱温为40 ℃,流速为0.3 mL/min, A相为水溶液,B相为乙腈,A、B相中均加入0.1%的甲酸;流动相梯度洗脱条件:0~0.5 min, 2%B; 0.5~10 min, 2%B~98%B; 10~16 min, 98%B; 16~18 min, 98%B~2%B; 18~20 min, 2%B。进样量为1 μL。

质谱条件 采用电喷雾电离(ESI)源与液相色谱系统连接并使用正离子模式(Pos)和负离子模式(Neg)进行数据采集;一级质谱全扫描范围*m/z* 70~1050;分辨率120000;辅助气流速30 L/min;喷雾电压3500 V (+)/3200 V (-);射频电压:70 V;毛细管温度:350 ℃;辅助气温度:300 ℃。二级质谱扫描(Full MS/dd-MS^2^)参数如下:扫描范围为*m/z* 70~1050;分辨率60000;隔离窗口*m/z* 1.5;碰撞能量20、40、60 eV。

#### 1.3.2 质谱成像法

采用配备AFADESI-MSI的质谱仪在正、负模式下进行数据采集,扫描范围为*m/z* 75~1050。毛细管电压为3500 V (+)/3200 V (-),离子源温度为350 ℃,氮气流速为0.6 L/min。喷雾溶剂为80%乙腈水溶液,流速为5 μL/min。扫描速度为200 μm/s,分辨率(X and Y pixel sizes)为150 μm。

### 1.4 数据分析

代谢组数据处理:质谱数据采用Compound Discoverer Metabolomics 3.3.1软件(Thermo Fisher公司)进行处理。采用单变量统计分析和主成分分析(principal component analysis, PCA)对样品进行统计分析。本实验以同时满足差异显著性(*p*-value<0.05和差异倍数(fold change>2)作为显著性差异代谢物的筛选标准。为了更直观显示样品间的关系以及代谢物在不同样品中的表达模式差异,将所有样品及差异代谢物以火山图形式展示。并对差异性代谢物进行京都基因和基因组数据库(Kyoto Encyclopedia of Genes and Genomes, KEGG)(http://www.kegg.jp/)通路富集分析。

质谱成像数据处理:将原始数据(.raw)格式转换为.cdf格式。将.cdf格式数据导入MassImage Pro软件,对数据进行图像重构,并进行背景扣除,容差设置为0.005 Da,提取目标离子进行成像。

## 2 结果与讨论

### 2.1 实验数据质量评价

QC样品在不同采集模式下的总离子流图如图1所示。为考察方法的稳定性,在分析过程中穿插6个QC样品。通过Compound Discovery对QC样品进行峰提取、峰对齐、化合物质量亏损过滤、化合物搜库,并根据二级谱图与标准谱库比对进行化合物检索鉴定。Compound Discovery进行峰对齐后显示,在样品分析过程中各色谱峰的保留时间相对标准偏差为0.04%~0.51%, QC样品峰强度相对标准偏差为2.3%~7.1%,表明本实验方法具有良好的重复性和稳定性。

**图1:**
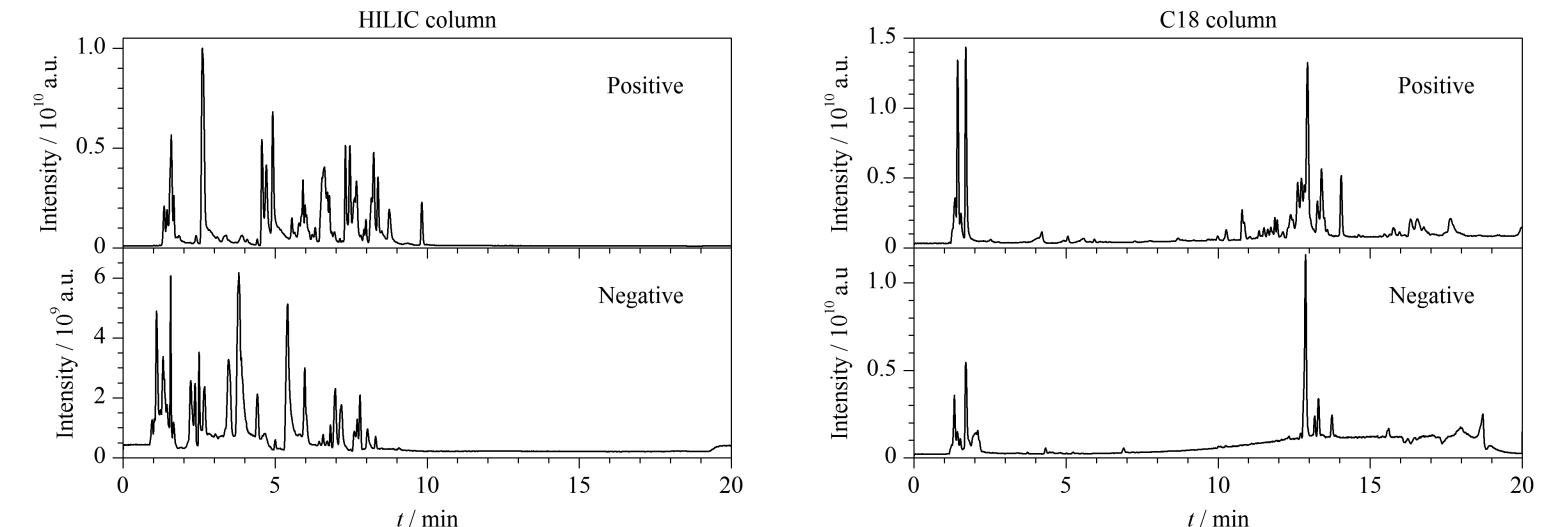
QC样品在不同色谱柱以及不同扫描模式下的总离子流图

对Compound Discovery鉴定到的化合物进行筛选,为提高准确性,剔除掉mzCloud匹配度小于70分的物质后,本实验在C18柱正离子模式下鉴定到384种物质,C18柱负离子模式下鉴定到196种物质。HILIC柱正离子模式下鉴定到227种物质,HILIC柱负离子模式下鉴定到148种物质(图2a)。对4种模式下鉴定到的化合物进行合并去重后,共获得759种化合物(图2b)。进一步进行PCA分析(图2c~f),其中QC样品的聚集程度反映了实验的重复性。结果表明4种模式下QC样品均紧密聚集在一起,进一步表明本次试验的数据稳定可靠,且从PCA结果可以看出烟碱处理组和盐水组样品间具有较好的区分度。

**图2:**
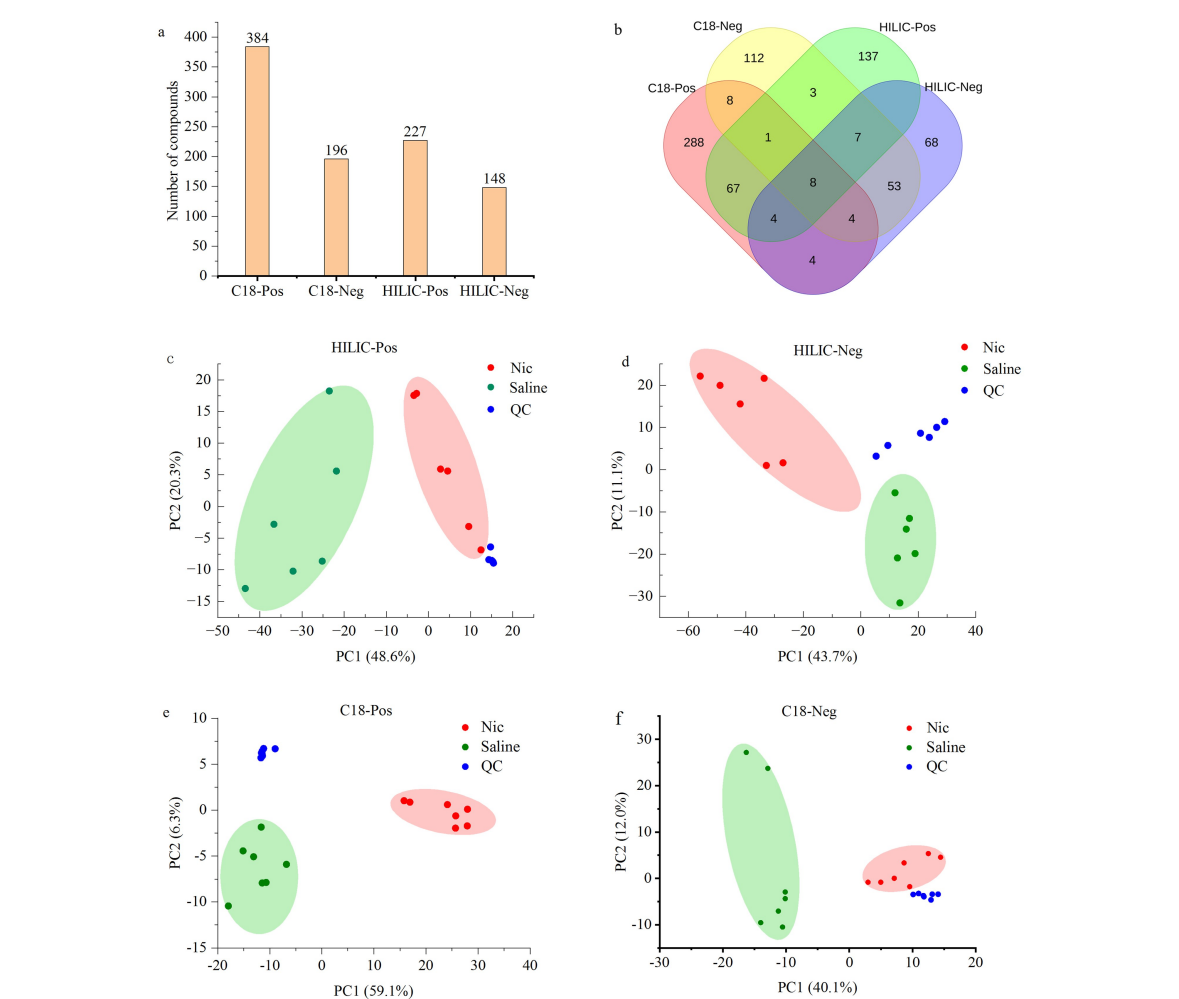
采用不同色谱柱及不同电离模式下获得的代谢组数据分析

### 2.2 单变量统计分析

基于单变量分析,对各分离及采集模式下鉴定到的mzCloud匹配度大于70分的物质进行差异分析,采用火山图的形式进行可视化展示|log_2_ FC|>1及*p*-value<0.05的差异代谢物,结果如图3所示。

**图3:**
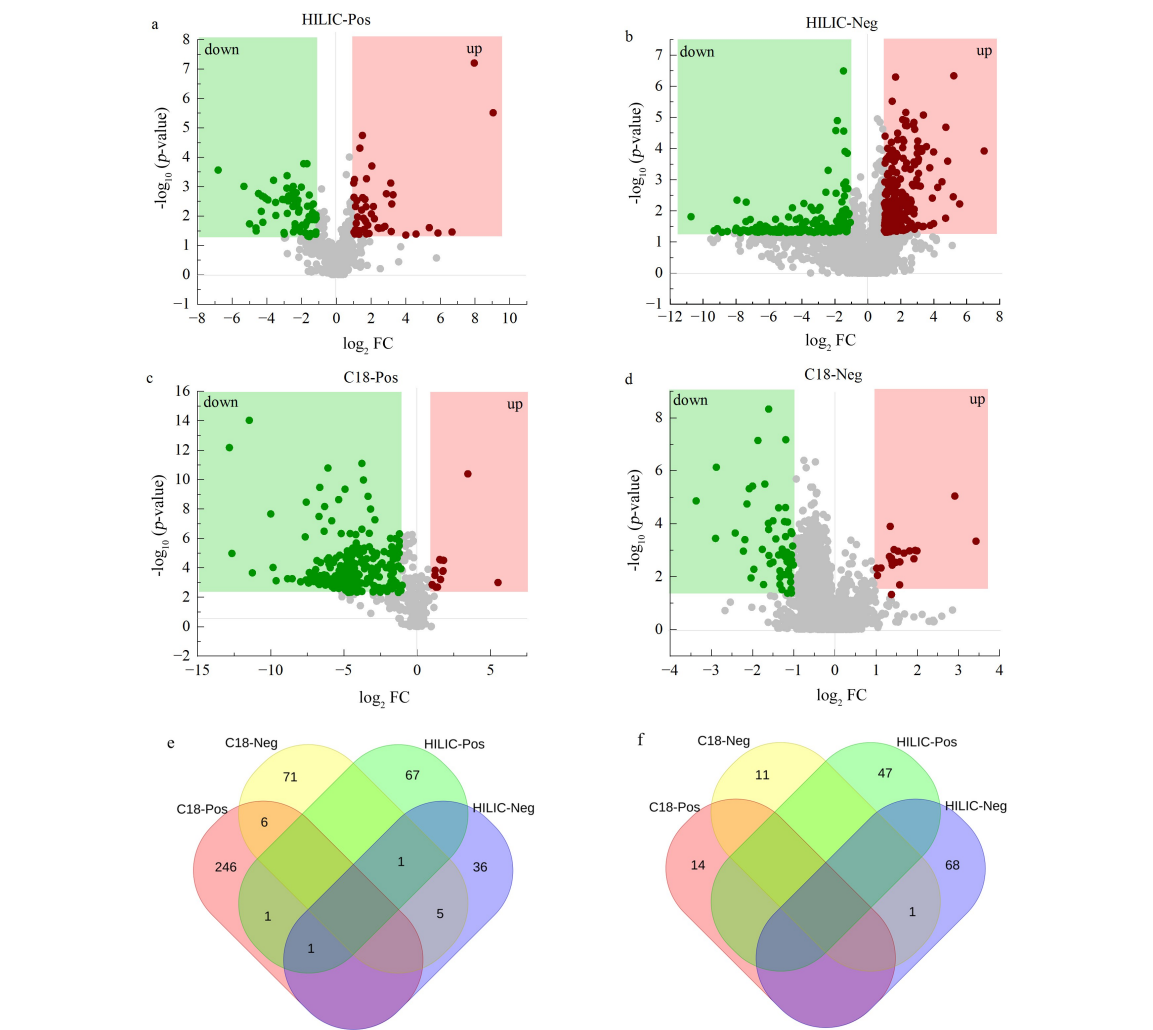
不同扫描模式下的代谢物差异性分析

结果表明,HILIC柱正离子模式下共获得122个显著性差异代谢物,其中74个代谢物下调,48个代谢物上调,HILIC柱负离子模式下共获得127个显著性差异代谢物,其中48个代谢物下调,79个代谢物上调。C18柱正离子模式下共获得271个显著性差异代谢物,其中257个代谢物下调,14个代谢物上调,C18柱负离子模式下共获得105个显著性差异代谢物,其中92个代谢物下调,13个代谢物上调。对不同采集模式下鉴定到的差异代谢物进行合并、去重,共获得575个显著性差异代谢物,其中434个代谢物下调(图3e), 141个代谢物上调(图3f)。

### 2.3 差异代谢物的KEGG通路富集分析

采用MetaboAnalyst在线分析软件对上一步筛选到的差异性代谢物进行KEGG代谢通路富集分析,结果如图4所示。

**图4:**
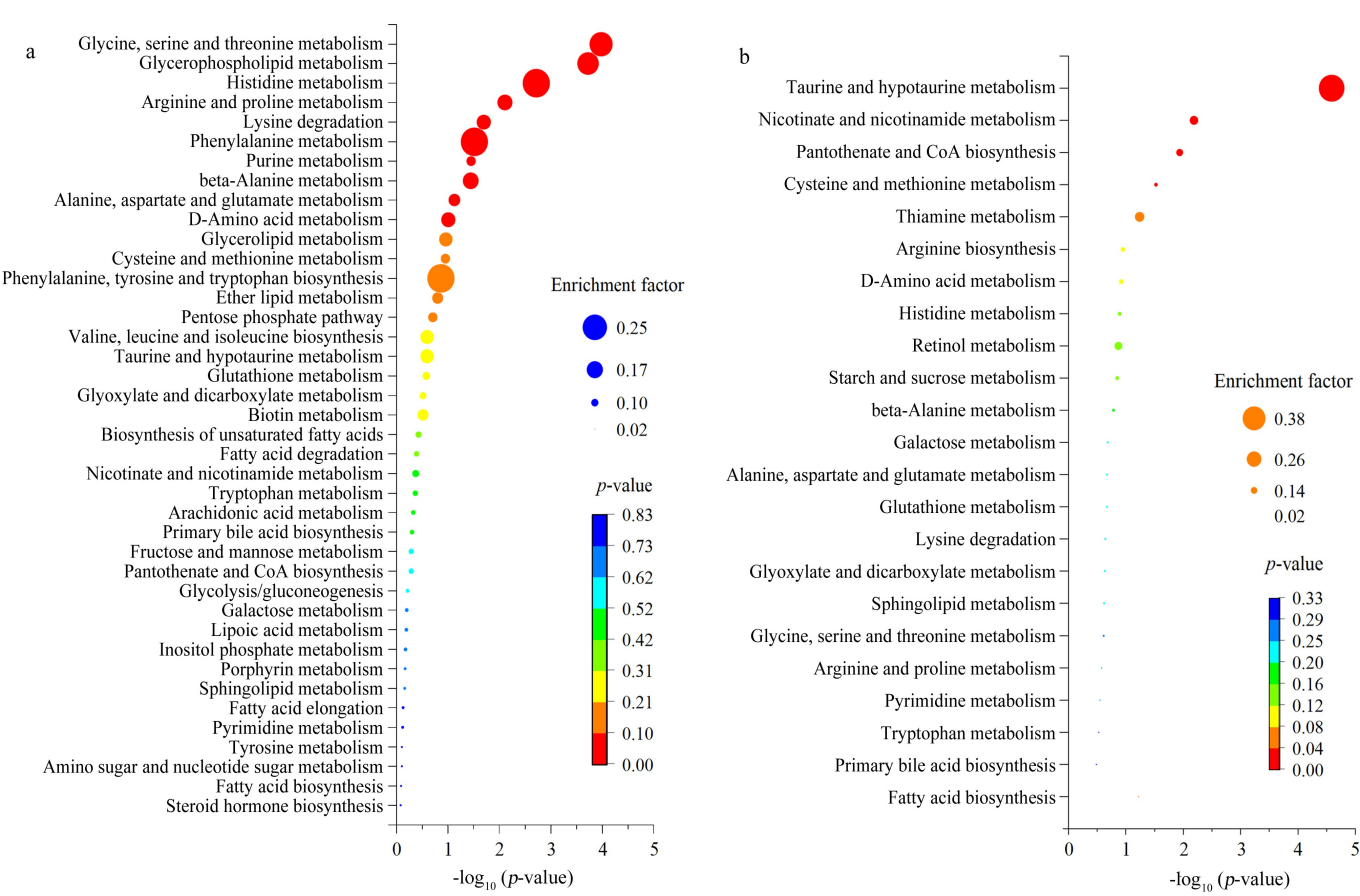
KEGG富集通路气泡图

以KEGG代谢通路大类分类,主要影响到机体必需氨基酸代谢、脂质代谢、核苷酸代谢、碳水化合物代谢、辅因子和维生素代谢以及其他氨基酸代谢6大类,共涉及42条代谢通路;其中,氨基酸代谢包括半胱氨酸和甲硫氨酸代谢,精氨酸生物合成,组氨酸代谢,丙氨酸、天冬氨酸、谷氨酸代谢,赖氨酸降解,甘氨酸、丝氨酸和苏氨酸代谢,精氨酸和脯氨酸代谢,色氨酸代谢,苯丙氨酸代谢,苯丙氨酸、酪氨酸和色氨酸生物合成,缬氨酸、亮氨酸和异亮氨酸生物合成;脂质代谢包括鞘脂代谢,初级胆汁酸生物合成,甘油酯代谢,甘油磷脂代谢,醚酯代谢,不饱和脂肪酸生物合成,脂肪酸降解,花生四烯酸代谢,脂肪酸延长,脂肪酸生物合成,类固醇激素生物合成;核苷酸代谢主要影响嘧啶代谢;碳水化合物代谢包括淀粉和蔗糖代谢,半乳糖代谢,乙醛酸和二羧酸代谢,戊糖磷酸途径,果糖和甘露糖代谢,糖酵解/糖异生,肌醇磷酸代谢,氨基酸和核苷酸糖代谢;辅因子和维生素代谢包括烟酸和烟酰胺代谢,泛酸和辅酶A生物合成,硫胺代谢,视黄醇代谢,生物素代谢,脂肪酸代谢,卟啉代谢;其他氨基酸代谢包括牛磺酸和亚牛磺酸代谢,D-氨基酸代谢,*β*-丙氨酸代谢,谷胱甘肽代谢。

### 2.4 质谱成像法分析脑切片中差异性代谢物

在考察烟碱多次暴露对脑内内源性代谢物的影响前,我们首先研究了腹腔注射烟碱20 min后其在小鼠脑内的分布信息,结果如图5所示,可以看出,烟碱在脑内呈不均匀分布情况,其中皮层、丘脑和小脑中含量最高,纹状体、中脑次之,海马、下丘脑中含量较低,即皮层≈丘脑≈小脑≈纹状体>中脑>海马>下丘脑。进一步采用AFADESI-MSI更直观考察烟碱暴露后对脑内内源性代谢物的影响。在采用正、负离子数据采集后,我们对代谢组分析中富集到的代表性差异代谢物进行了可视化展示。结果如图6所示,烟碱暴露显著降低了全脑范围内胆碱(*m/z* 104.1072)和皮层区域谷氨酸(*m/z* 146.0444)含量;氨基酸代谢通路中天冬氨酸(*m/z* 132.0280)、丝氨酸(*m/z* 104.0346)在全脑内都降低,皮层中更为显著;脂质代谢通路中显著增加了海马、丘脑以及小脑灰质区乙酰基肉碱(*m/z* 204.1228)含量;核苷酸代谢通路中纹状体区腺苷(*m/z* 268.1028)、肌苷(*m/z* 267.0735)和黄嘌呤(*m/z* 151.0247)等均被显著下调;碳水化合物代谢途径中三羧酸循环中的苹果酸(*m/z* 133.0127)在全脑范围内均被显著下调;其他氨基酸代谢中皮层区域牛磺酸(*m/z* 124.0068)被显著抑制。

**图5:**
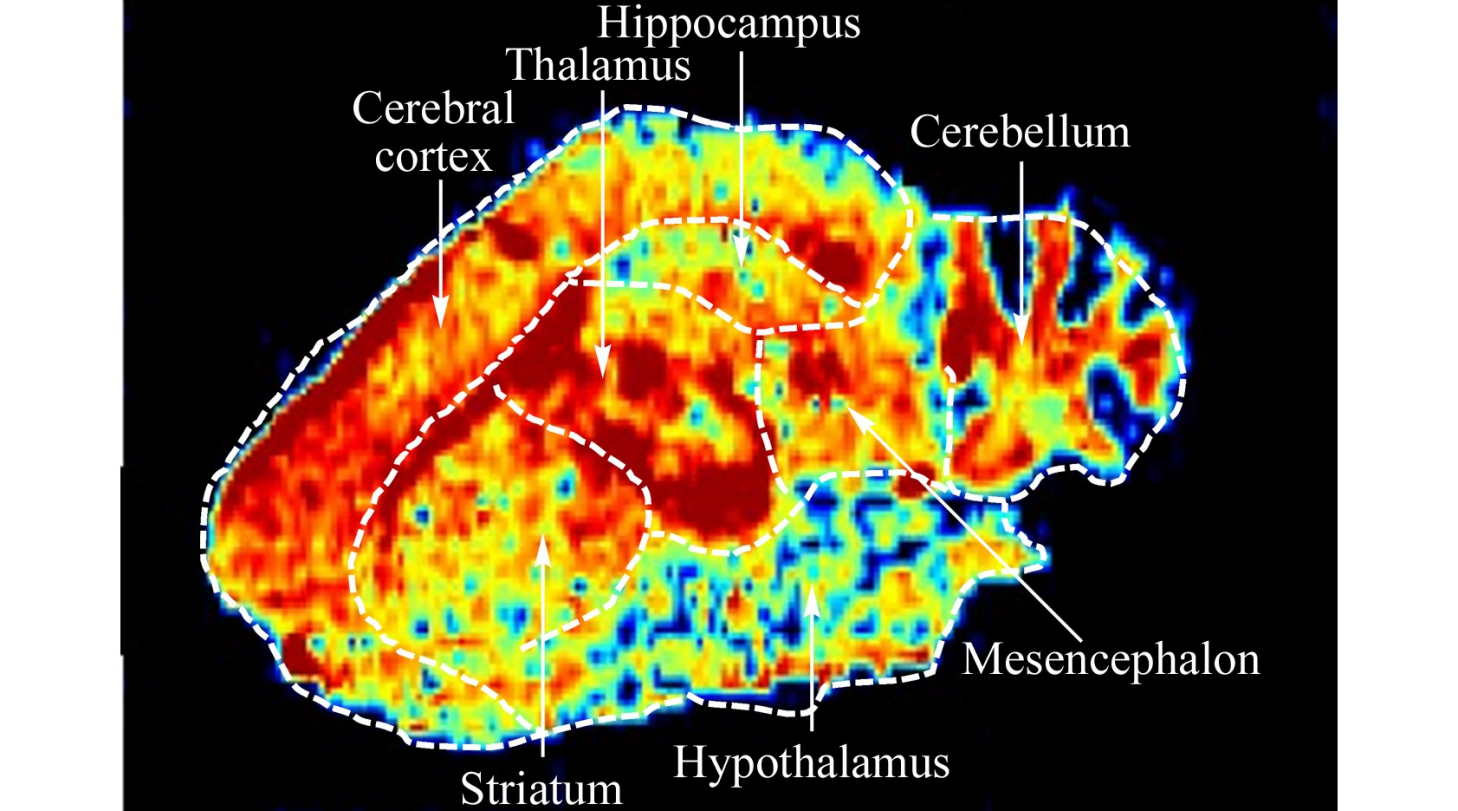
烟碱在小鼠脑内的分布特征

**图6:**
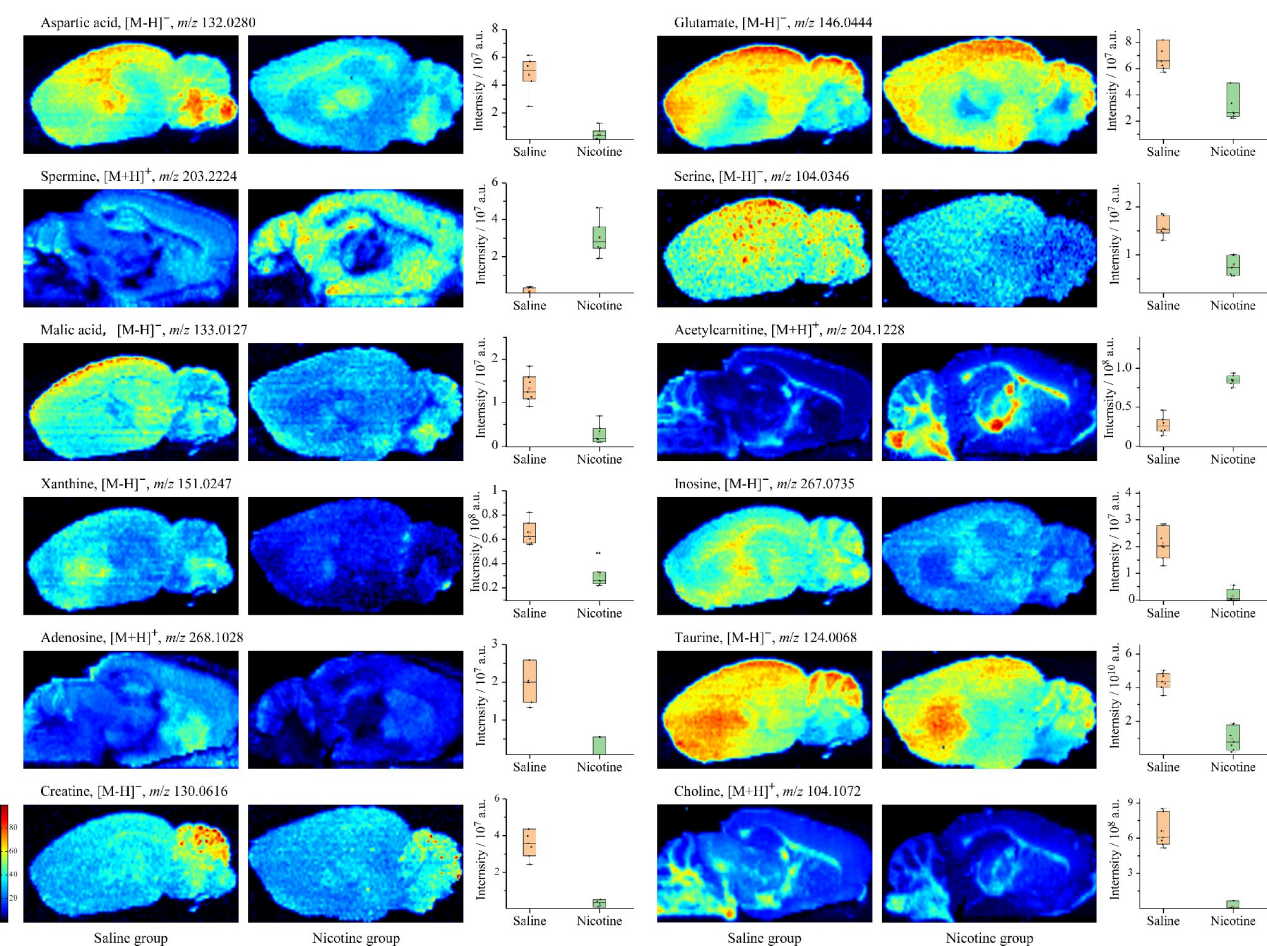
脑组织切片中烟碱暴露后诱导的代表性内源性代谢物的空间可视化结果

## 3 结论

本研究采用UHPLC-MS/MS代谢组学方法对烟碱暴露后小鼠脑内内源性代谢物的变化进行分析,共鉴定出575种差异性代谢物。通过KEGG对差异性代谢物进行富集分析,其中434个下调代谢产物富集到40条代谢通路,141个上调代谢产物富集到23条代谢通路。上调和下调代谢产物共涉及机体必需氨基酸代谢、脂质代谢、核苷酸代谢、碳水化合物代谢、辅因子和维生素代谢以及其他氨基酸代谢6大类42条代谢通路。通过AFADESI-MSI对代表性差异性代谢产物进行可视化,发现全脑范围内胆碱、丝氨酸、天冬氨酸及苹果酸含量都被显著下调,皮层区域牛磺酸、海马区域乙酰基肉碱以及纹状体区域的腺苷类物质均被显著影响。这些结果为进一步理解烟碱入脑引起的代谢物变化规律提供了新的实验依据。
